# Supplementary material for: The Mutagenic Plasticity of the Cholera Toxin B-Subunit Surface Residues: Stability and Affinity
Source: Toxins (Basel). 2024 Mar 4;16(3):133. doi: 10.3390/toxins16030133 (PMC10974167; doi:10.3390/toxins16030133)
Supplement: Supplementary file 1 [file toxins-16-00133-s001.zip › toxins-2876673-supplementary.pdf]

toxins

MDPI

Supplementary Materials: The Mutagenic Plasticity of the Cholera Toxin B-Subunit Surface Residues: Stability and Affinity

Cheuk W. Au, Iain Manfield, Michael E. Webb, Emanuele Paci, W. Bruce Turnbull and James F. Ross

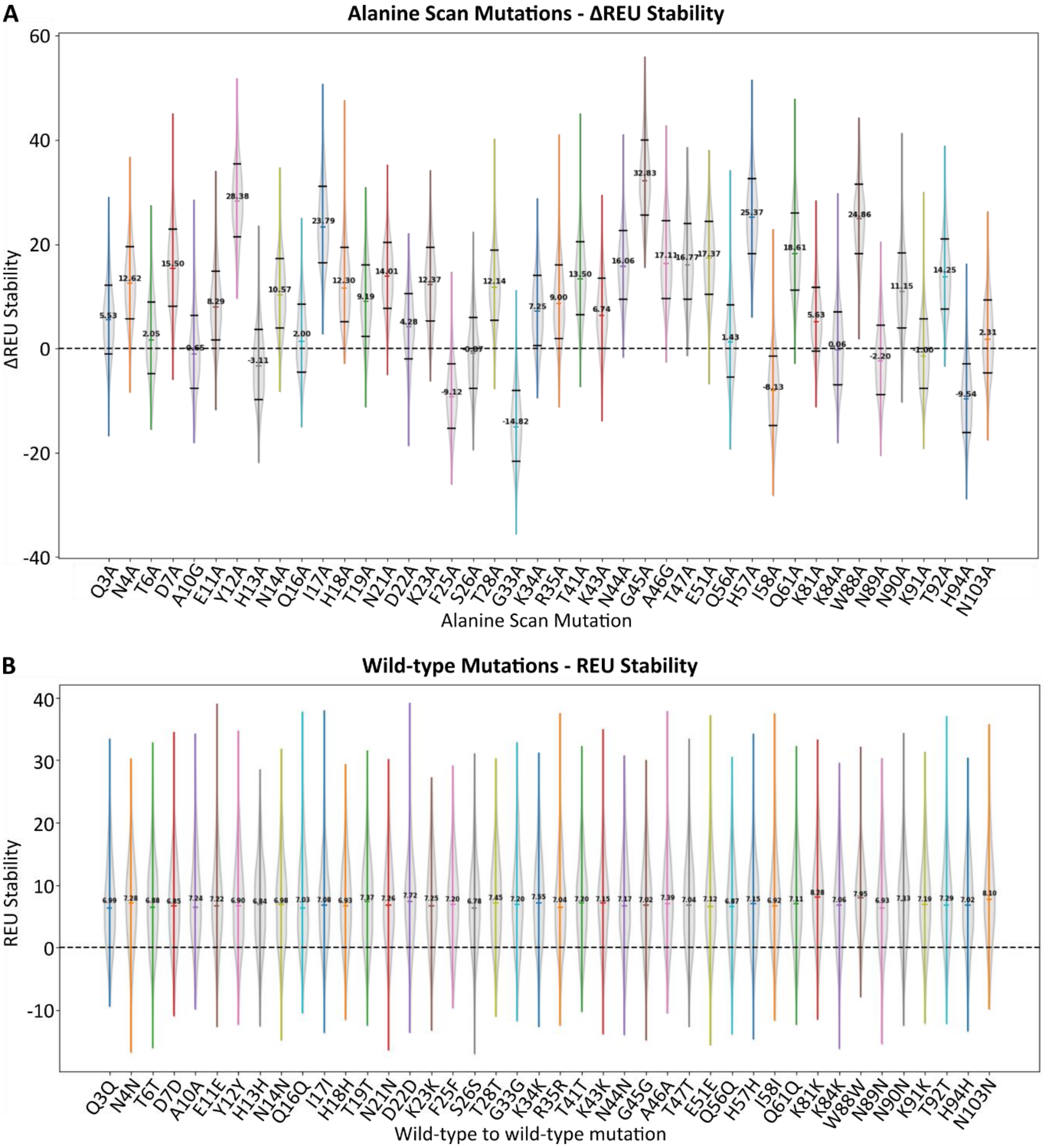

Figure S1. Rosetta energy score distributions for a 42 residue alanine scan of surface positions of CTB. A) 1000 wt to mutant structures generated per position. In this experiment, the wt amino acid is replaced with alanine (or glycine if wt-amino acid is alanine) and the ΔREU is calculated, where ΔREU is the mutant median total_score minus the median of the wt to wt total_score. **B)** 1000 wt to wt mutation structures generated per position (effectively as relaxations). In this experiment, the amino acid is

flagged for mutation to the wild-type residue, as a negative control, showing the energy distributions for relaxations of the starting structure. .

Note: The energy distribution above for B) have a non-zero median score as the starting structure was the lowest energy pose in the initial relaxation. As all modelling uses the initial pre-relaxed structure, each data set show a redistribution of stabilities from that starting pose.

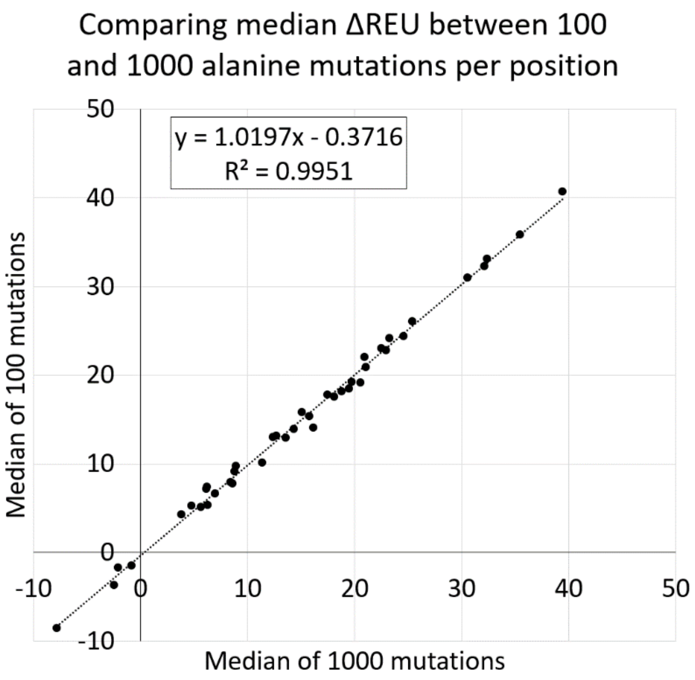

Figure S2. Comparing median ΔREU between 1000 mutations and 100 mutations. To complete the saturation scan in a timely manner it was necessary to reduce the number of structures assessed for each mutant. Here we provide a comparison of the median ΔREU values used in the assessment of energies between the 1000x data set (from the alanine scan, Figure S1) and 100x data set (from the alanine's of the saturation scan, Figure 3).

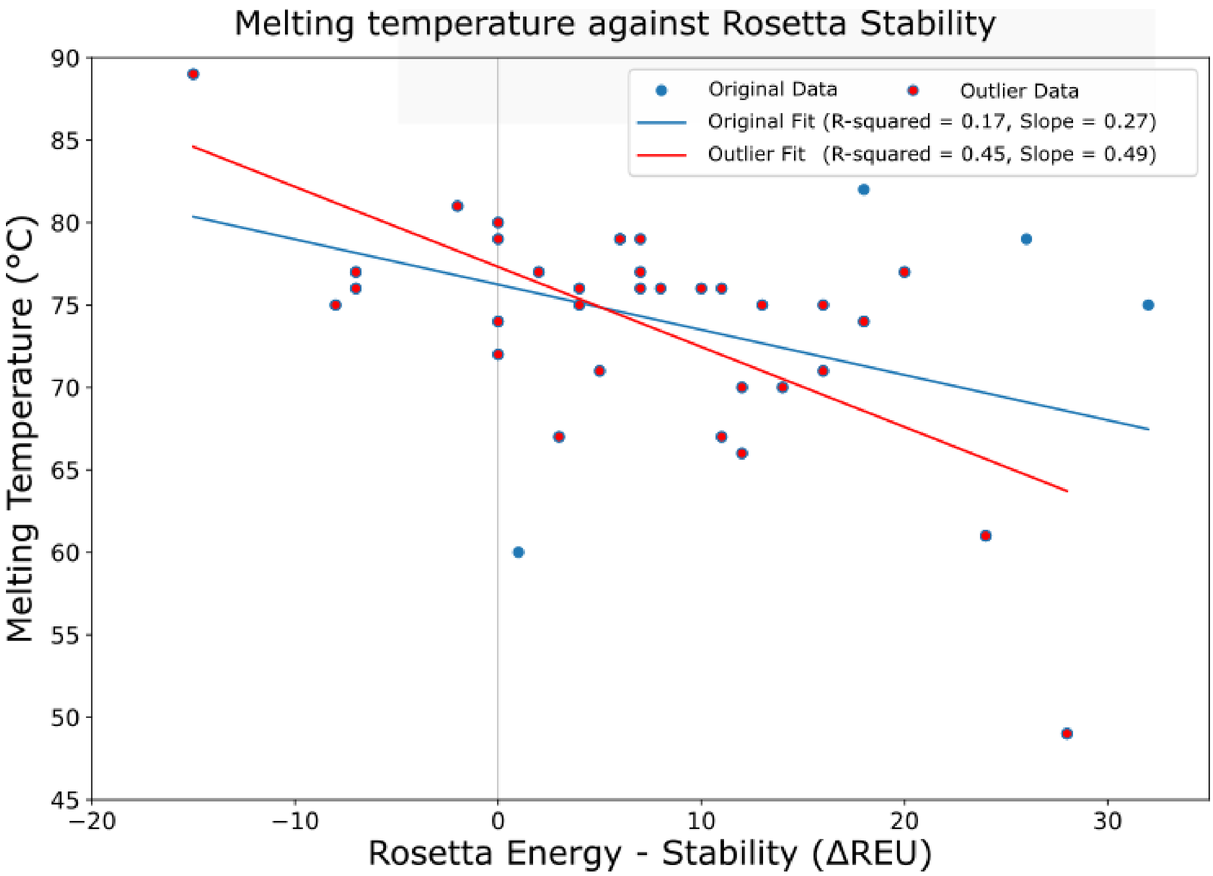

Figure S3. Assessing the relationship between melting temperature and Rosetta stability score. The fit between the computationally derived Rosetta Stability ('total_score' in Rosetta Energy Units minus the 'total_score' of the WT pose) against the melting temperature determined by differential scanning fluorimetry. The 'Original' data set is in blue and the 'Outlier' dataset in red, from which four outliers have been removed.

Note: To remove outliers, the R-squared was recalculated in the absence of each data point and ranked by improvement in R-squared. The data point which, when removed, gives the largest increase in R-squared was classed as an outlier and permanently removed from the dataset. This was conducted four times, representing ~ 10 % of the dataset.

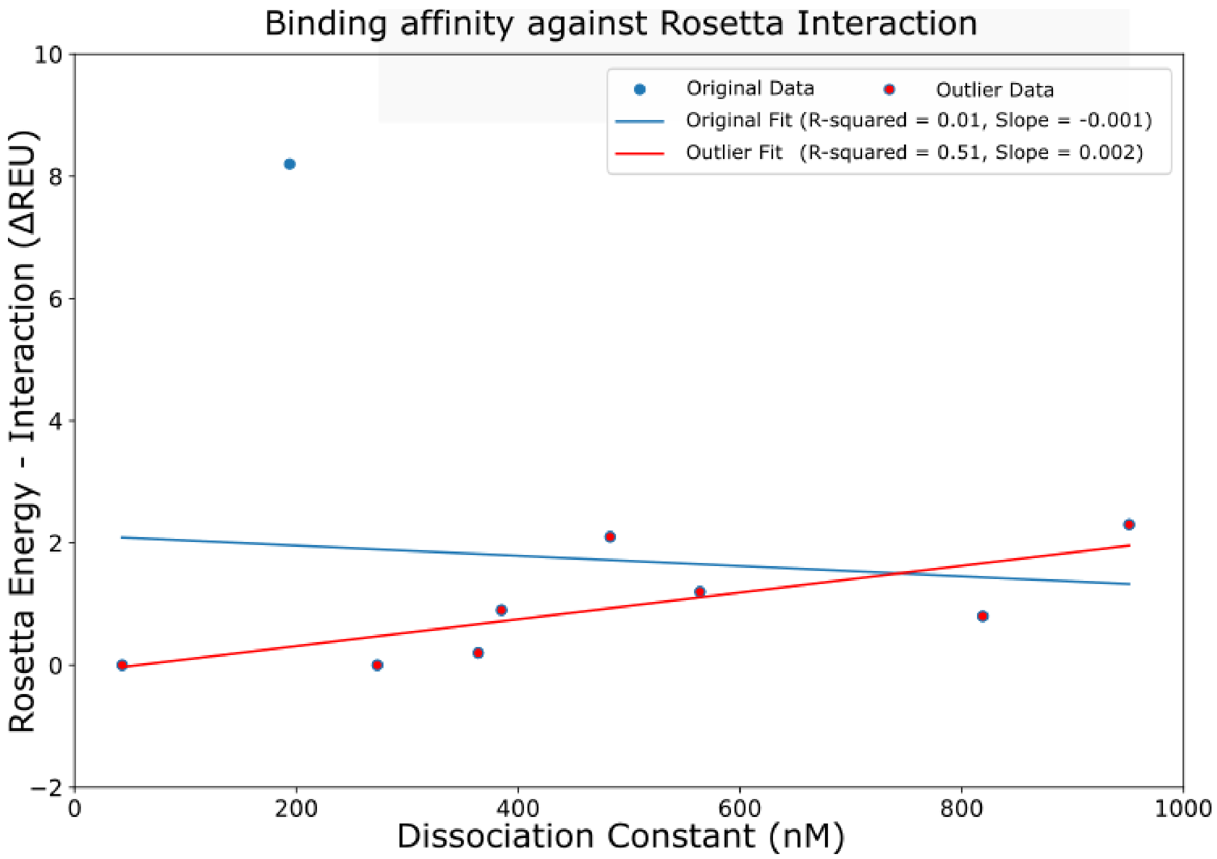

Figure S4. Assessing the relationship between the dissociation constant and Rosetta interaction score. The fit between the computationally derived Rosetta Interaction between CTB and GM1 ('dg_separated' in Rosetta Energy Units minus the 'dg_separated' of the WT interaction) against the dissociation constant determined by isothermal titration calorimetry. The 'Original' data set is in blue and the 'Outlier' dataset in red, from which one outlier has been removed.

Note: To remove outliers, the R-squared was recalculated in the absence of each data point and ranked by improvement in R-squared. The data point which, when removed, gives the largest increase in R-squared was classed as an outlier and permanently removed from the dataset. This was conducted once, representing ~ 10 % of the dataset.

resi	K	H	R	E	D	Q	N	T	S	G	C	P	A	V	I	L	M	F	Y	W
1	-4	-3	1	-3	-10	-5	-10	-1	-2	-4	4	-2	-5	1	0	-3	-4	9	1	6
3	1	2	0	2	11	1	0	-8	-3	16	9	20	7	10	10	4	6	3	9	9
4	15	24	10	9	-2	13	0	10	4	7	9	105	12	22	33	22	24	41	33	32
6	0	6	6	3	-21	11	6	1	1	7	12	118	4	-1	4	9	8	12	9	14
7	12	7	25	-8	1	-7	19	17	22	31	28	105	16	16	9	10	15	26	21	23
10	-2	0	-7	3	5	-2	-4	3	-5	9	3	92	0	9	11	-6	4	4	0	2
11	11	15	14	-1	12	0	8	10	3	17	14	52	7	33	15	5	6	20	16	21
12	6	29	12	18	21	12	11	15	24	31	19	87	28	8	7	-5	12	-2	-1	24
13	-5	1	0	-7	-12	-5	-6	-4	-8	-1	8	-18	-4	7	7	-2	5	7	4	11
14	5	2	12	6	-2	9	-2	25	6	-3	19	141	8	69	75	12	15	9	7	16
16	4	9	10	3	9	1	11	6	4	10	12	40	2	13	13	13	14	11	10	21
17	25	22	23	-1	17	10	18	10	20	35	22	21	24	4	-1	-6	26	17	25	30
18	15	2	20	14	-2	11	7	9	15	35	14	27	14	-7	-8	9	16	4	-2	18
19	2	5	3	-3	-5	2	2	-1	6	18	11	-4	7	-7	-6	-3	6	0	0	5
20	33	4	60	12	19	5	13	15	28	32	18	25	22	-3	-9	1	15	-3	9	42
21	1	10	6	6	-5	6	-1	21	9	10	16	1304	13	68	73	13	20	23	11	22
22	12	8	5	4	0	-4	4	1	4	18	4	125	4	8	10	0	10	32	31	36
23	-2	7	1	15	14	8	14	4	8	16	11	14	11	7	7	10	13	12	7	26
25	-11	-1	1	-1	3	-1	5	5	5	11	9	33	-7	-3	0	-2	3	3	-2	7
26	0	11	1	9	16	1	23	5	1	20	9	126	-1	15	15	12	9	18	26	27
28	22	6	12	-1	-4	8	17	-3	17	29	7	63	10	-19	-18	4	5	4	5	49
33	-7	-4	-9	-16	-10	-9	1	-10	-12	-2	-6	-26	-15	-6	-2	-6	-4	4	-4	1
34	0	0	4	1	-4	4	-7	18	2	-16	16	435	6	66	69	6	13	3	3	11
35	19	19	-1	33	32	15	12	16	18	24	26	97	8	18	31	23	26	32	31	47
36	26	13	20	1	-8	18	5	9	12	20	9	17	3	1	4	-7	8	5	14	11
41	7	0	-1	6	17	-2	11	0	6	24	15	71	12	-2	-1	8	6	9	17	17
42	58	28	48	44	38	36	31	30	36	40	34	61	29	20	29	19	26	-1	1	49
43	0	-1	-1	3	4	4	-4	4	-4	12	13	-14	6	10	15	3	5	0	0	1
44	3	12	5	12	-1	6	1	13	4	18	14	71	16	13	19	10	18	21	15	16
45	22	22	29	27	25	17	18	39	25	0	38	402	32	92	91	30	33	30	29	37
46	7	16	11	2	6	7	7	0	6	18	10	66	1	13	15	6	14	25	26	27
47	9	9	3	23	29	16	18	1	11	33	14	22	18	-4	-8	18	21	5	3	25
48	58	16	44	41	41	37	33	28	30	50	47	60	23	29	68	37	35	0	-3	41
49	18	25	29	13	28	-1	19	10	13	27	14	47	10	3	5	15	7	11	13	66
51	27	18	21	1	0	12	18	7	10	20	15	-12	18	14	22	0	16	24	28	36
52	24	27	21	35	42	21	31	17	20	34	16	15	18	0	7	14	23	37	27	35
53	23	25	30	19	29	26	28	22	16	32	26	-1	18	22	17	34	30	29	26	36
54	4	5	1	4	-10	5	-16	-12	-10	1	0	33	4	1	-2	4	7	18	9	23
55	5	6	11	2	0	6	4	9	0	10	19	-9	7	15	18	11	15	12	4	10
56	-4	3	3	-8	-5	-1	-4	-2	-6	4	7	62	0	4	6	1	6	7	4	10
57	12	-1	26	27	14	22	14	10	19	31	24	104	26	15	14	15	19	6	6	18
58	-9	-14	0	-19	-21	-8	-12	-10	-15	-2	0	11	-7	1	0	-10	-3	-4	-2	3
59	12	18	18	2	1	14	12	14	13	20	28	-1	13	19	20	19	22	22	16	19
60	-6	4	-9	-11	-10	0	-3	-1	0	10	10	68	1	2	-6	-8	2	2	5	3
61	31	44	25	18	34	0	35	16	20	35	25	121	20	12	19	17	16	49	41	37
62	0	19	12	10	11	10	11	15	15	23	20	84	12	10	9	7	13	19	17	22
79	10	3	18	1	-1	-1	2	24	7	3	20	335	11	82	84	18	22	14	16	22
81	0	7	1	11	6	2	7	0	7	13	13	3	5	-2	-5	10	5	9	5	11
83	2	-5	2	0	-5	0	8	7	4	16	14	33	-1	4	-1	-4	14	8	3	8
84	1	2	11	2	18	3	5	15	8	19	20	143	1	10	14	3	1	3	1	21
88	8	15	21	10	8	17	9	21	24	38	25	48	26	13	11	16	16	9	2	1
89	-1	21	0	6	7	-1	0	-6	0	11	8	27	-2	-7	-6	-7	-4	27	12	18
90	-1	5	-3	3	-2	-4	1	-1	4	13	10	52	11	-2	-3	-3	9	9	5	18
91	0	5	-3	10	17	1	9	1	5	21	9	115	0	-1	2	7	11	0	-6	-1
92	6	5	-1	0	-15	2	-14	2	5	43	24	19	16	-3	-2	5	12	4	3	23
94	1	3	1	-4	-1	-9	1	17	-3	11	15	-31	-8	6	22	-7	-2	5	1	12
102	13	24	8	27	34	12	22	10	1	16	10	13	1	2	-22	13	19	16	21	27
103	3	3	5	-5	-3	-7	1	4	-3	6	11	68	3	5	5	-2	4	-3	-3	8

Figure S5. ΔREU median stability energy values for the computational saturation scan without ligand. ΔREU stability estimates vs the wild-type stability for each amino acid at each position under study.

**with ligand**	stability																			
**resi**	K	H	R	E	D	Q	N	T	S	G	C	P	A	V	I	L	M	F	Y	W
**11**	17	10	15	2	8	7	17	23	17	21	23	61	11	21	25	10	12	21	21	27
**12**	47	10	38	37	42	31	28	32	39	31	32	61	32	32	20	1	17	-3	-1	25
**13**	-5	0	-1	-4	-5	2	1	5	6	5	6	39	0	4	6	-4	0	4	5	9
**14**	12	10	18	24	12	22	2	44	20	9	24	130	15	40	58	10	25	18	19	18
**33**	20	17	20	15	18	13	13	15	-2	0	10	-5	-16	10	19	21	6	13	19	19
**34**	3	1	0	4	0	9	-3	25	4	-30	11	233	0	59	47	-2	7	7	9	5
**35**	37	35	0	48	33	30	23	29	30	25	25	132	17	21	36	22	29	35	39	46
**51**	38	22	31	3	12	19	25	11	19	28	28	5	24	31	31	6	22	47	46	48
**55**	7	2	12	-2	3	7	8	7	1	15	25	11	12	20	17	9	14	19	15	7
**56**	4	-5	9	4	6	-1	8	3	5	15	18	41	10	10	13	1	10	10	12	2
**57**	30	0	38	33	20	30	15	17	31	23	31	84	29	26	28	17	23	13	11	19
**58**	-5	-8	-2	-15	-23	-7	-10	-9	-6	-4	2	18	-6	-1	1	-10	-4	6	8	5
**61**	41	18	33	8	15	1	18	21	19	28	21	78	15	20	16	14	8	28	24	24
**88**	59	26	54	51	45	57	34	53	48	54	45	69	40	43	46	52	44	15	18	0
**90**	7	21	6	17	10	8	2	4	14	21	25	57	17	8	13	8	10	32	37	27
**91**	-3	-5	-9	1	1	-2	6	-8	5	11	0	53	0	-5	2	-5	-7	6	12	-4

**with ligand**	interaction																			
**resi**	K	H	R	E	D	Q	N	T	S	G	C	P	A	V	I	L	M	F	Y	W
11	9	9	9	1	3	4	7	9	8	8	8	8	8	7	7	8	9	8	9	11
12	14	8	13	11	13	11	12	10	10	6	12	14	12	9	12	9	7	2	-1	11
13	-1	0	-1	1	0	2	0	-1	0	0	-1	7	2	0	-1	2	-1	-1	-1	0
14	-1	0	-2	-1	1	-2	0	0	0	0	-1	-1	1	0	-1	-3	0	-1	0	0
33	4	-4	1	5	5	0	3	4	2	0	1	0	-3	4	4	1	-1	-9	0	-4
34	1	1	-1	0	1	0	1	2	-1	-2	0	1	0	2	1	-2	1	-1	-1	-3
35	10	10	0	7	8	5	7	5	6	6	5	1	6	6	3	4	6	5	8	3
51	1	2	1	0	0	-1	2	4	3	3	3	0	2	3	3	0	2	1	3	4
55	0	0	1	0	0	-1	-1	-1	-1	0	0	1	0	1	-1	0	1	0	0	0
56	0	1	1	0	0	1	1	1	1	2	1	1	1	1	1	1	1	0	0	0
57	-2	-1	-2	0	0	-2	-2	-1	-2	0	0	-4	-3	-2	-3	-2	-3	-2	-2	-3
58	1	2	1	-3	-3	-1	0	1	2	1	0	2	1	0	0	-1	-2	-2	-1	0
61	6	3	2	2	-2	0	-2	-2	-3	-1	-1	2	0	-3	-1	0	0	1	-3	1
88	8	3	6	4	4	4	3	4	3	1	2	4	2	6	8	6	4	-1	1	0
90	1	1	1	-1	0	1	1	1	2	2	3	2	2	2	3	-1	1	3	1	0
91	1	-3	1	0	0	0	2	1	0	3	1	2	1	1	3	2	1	-2	0	2

Figure S6. ΔREU median stability and ΔREU median interaction energies for the computational saturation scan with ligand. ΔREU stability 'total_score' (mutant minus wild-type energies), and ΔREU interaction 'dg_separated' (mutant minus wild-type energies), for each amino acid at each position under study with the GM1 oligosaccharide bound.

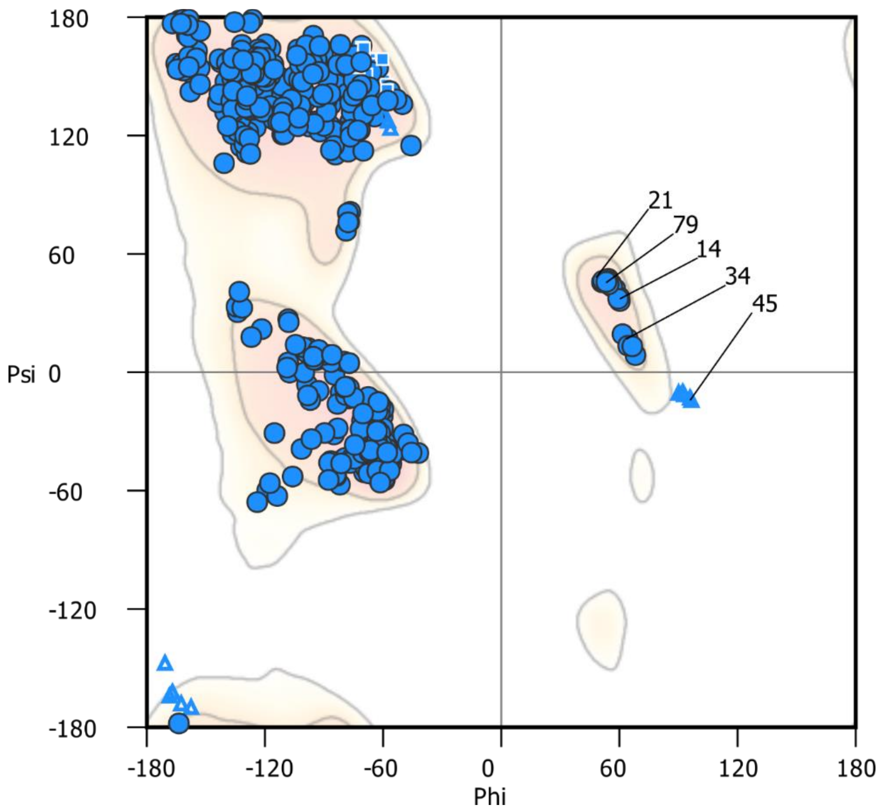

Figure S7. Ramachandran plot of relaxed 'symmetrised' CTB structure derived from pdb-2CHB. Prolines in squares, glycine's in triangles, other residues in circles. Density contours appropriate for non-glycine, non-proline residues. Highlighted residues 21, 79, 14, 34 and 45 have phi angles in the +50 to +110 range. This image was generated using coot.[1]

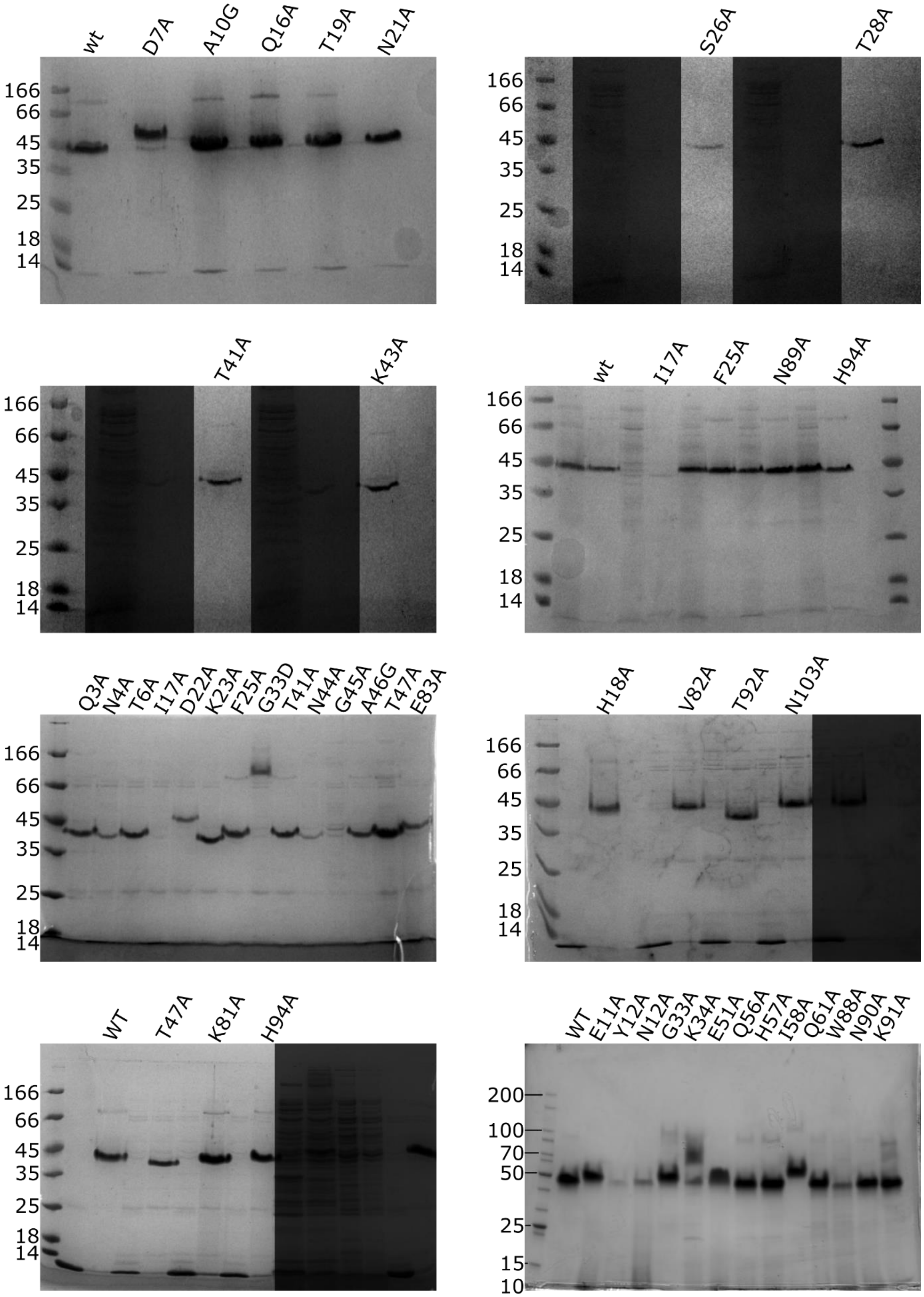

Figure S8. SDS PAGE of CTB mutants. SDS PAGE of expressed alanine mutants, SDS PAGE was conducted with and without boiling samples in loading dye, as a result CTB runs as protomer or pentamer respectively. Some lanes obscured as irrelevant. .

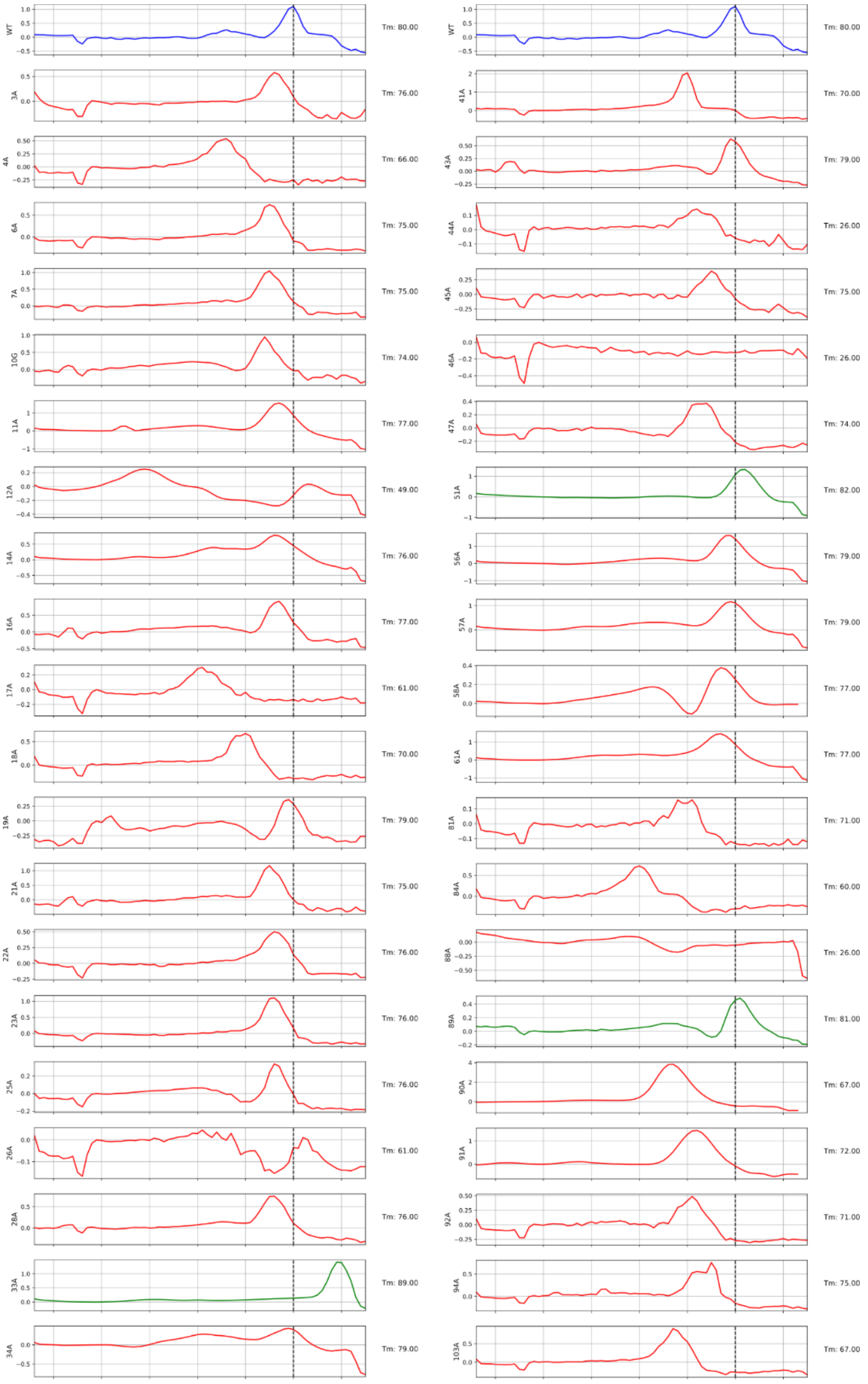

Figure S9. Raw differential scanning fluorimetry data. Un-normalised differential scanning fluorimetry data. Wild type in blue (data copied for convenience), Melting temperature greater than wild-type in green, less than wild-type in red.

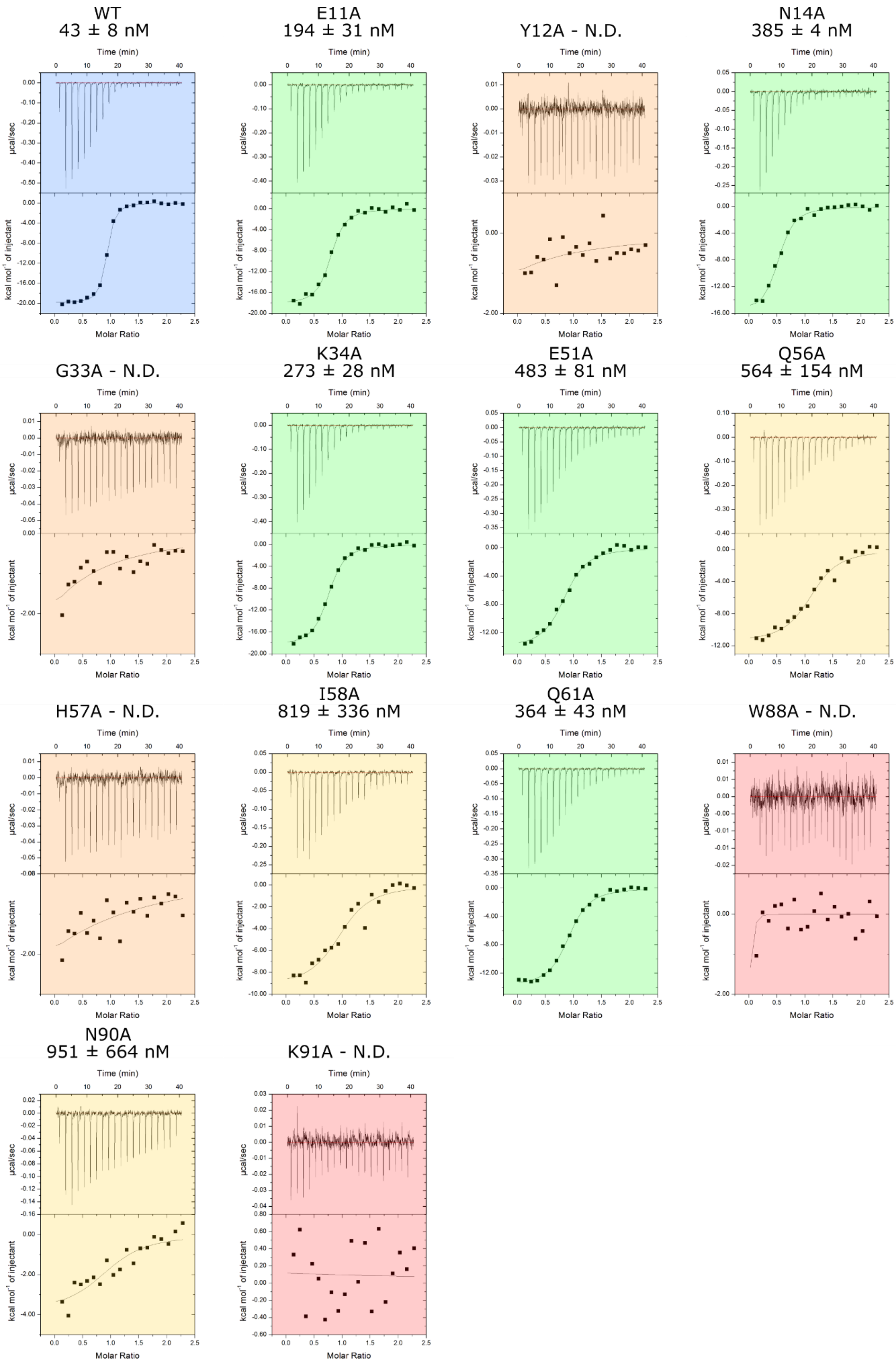
WT
43 ± 8 nM
E11A
194 ± 31 nM
Y12A - N.D.
N14A
385 ± 4 nM
G33A - N.D.
K34A
273 ± 28 nM
E51A
483 ± 81 nM
Q56A
564 ± 154 nM
H57A - N.D.
I58A
819 ± 336 nM
Q61A
364 ± 43 nM
W88A - N.D.
N90A
951 ± 664 nM
K91A - N.D.
Time (min)
µcal/sec
kcal mol⁻¹ of injectant
Molar Ratio

Figure S10. ITC binding affinities of alanine mutants. The binding curves have been loosely colour coded as follows. **Blue)** under 100 nM. **Green)** between 100 and 500 nM. **Yellow)** between 500 and 1000 nM, low confidence. **Orange)** Evidence of binding but low affinity (+1000 nM) and very low confidence. **Red)** no evidence of binding.

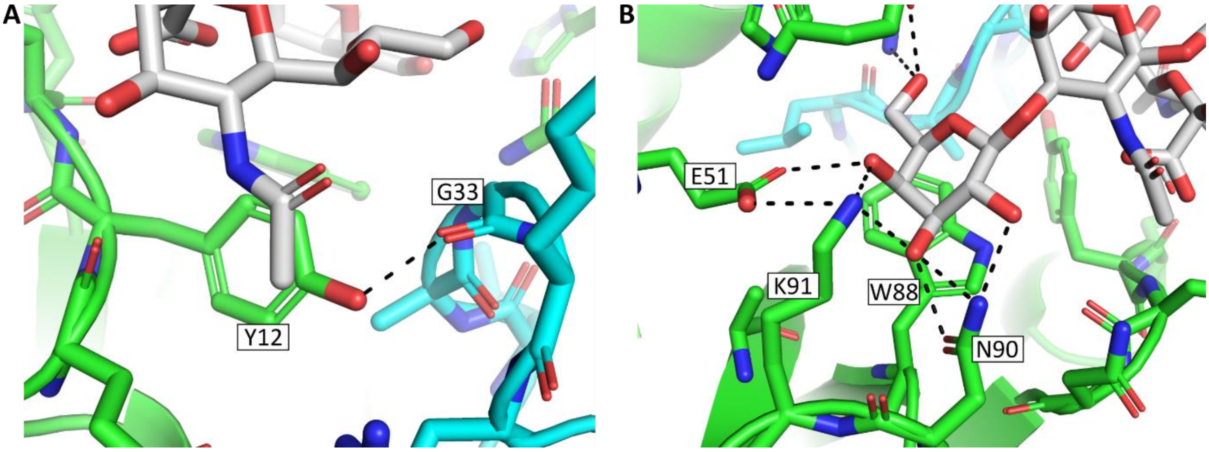

Figure S11. Molecular detail at the GM1-CTB interface. Protein chains in green and blue, GM1 in grey, potential hydrogen bonds in black. A) Focus in Y12 hydrogen bonding to G33. B) Focus on W88 CH-π interaction with GM1 and K91/E51 interaction with GM1 and each other.

Table S1: metrics from ITC of alanine scan mutants.

	Stoichiometry (n)	Kd (nM)	ΔH (kcal/mol)	ΔS (cal/mol/deg)
Wild-type	0.9 ± 0.0	43 ± 8	-2.0 ± 0.1	-33.3
E11A	0.7 ± 0.0	194 ± 31	-1.8 ± 0.4	-30.4
Y12A	0.1 ± 14	3000 ± 2.3E^{6}	-3.4 ± 5400	-92.8
N14A	0.5 ± 0.0	385 ± 4	-1.6 ± 0.6	-24.1
G33A	0.3 ± 2.7	2.0E^{5} ± 4.1E^{5}	-1.4 ± 150	-24.7
K34A	0.7 ± 0.0	273 ± 28	-1.8 ± 0.3	-32
E51A	0.8 ± 0.0	483 ± 81	-1.4 ± 0.4	-18.7
Q56A	1.1 ± 0.0	565 ± 155	-1.2 ± 0.5	-10.2
H57A	0.8 ± 5.5	3.0E^{4} ± 1.1E^{5}	-0.09 ± 73	-8.19
I58A	1.0 ± 0.0	820 ± 337	-9.3 ± 7.6	-3.38
Q61A	0.9 ± 0.0	365 ± 43	-1.4 ± 2.2	-17.1
W88A	0.1 ± 11.3	N.D.	-7.2 ± 190000	-1.1
N90A	1.0 ± 0.1	952 ± 664	-0.04 ± 7.2	13.8
K91A	1.7 ± 0.0	N.D.	1200 ± 1.1E9	3910
